# Direct In-Mold Impregnation of Glass Fiber Fabric by Polypropylene with Supercritical Nitrogen in Microcellular Injection Molding Process

**DOI:** 10.3390/polym15040875

**Published:** 2023-02-10

**Authors:** Qichao He, Weimin Yang, Jian Wang, Feng Ren, Da Wang, Fuhai Li, Zhonghe Shi

**Affiliations:** 1 College of Mechanical and Electrical Engineering, Beijing University of Chemical Technology, Beijing 100029, China; 2 State Key Laboratory of Organic-Inorganic Composites, Beijing University of Chemical Technology, Beijing 100029, China

**Keywords:** in-mold impregnation, microcellular injection molding, polypropylene, supercritical nitrogen, continuous fiber reinforced thermoplastics, glass fiber

## Abstract

Combining microcellular injection molding and insert injection molding, an injection molding technique for glass fiber fabric (GFF) reinforced polypropylene (PP) composite foams was proposed. The GFF was directly set in the mold cavity, and then the PP with supercritical nitrogen (SCN) was injected into the cavity for in-mold impregnation. The impregnation effects of two types of GFFs (EWR300 and EWR600) by the PP/SCF solutions at different injection temperatures (230, 240, and 250 °C) were investigated. The results of the morphological and tensile properties of the samples showed that the interfacial bonding was not good, because of the heterogeneity between the GFF and PP. In comparison with solid PP, the unfoamed GFF/PP did not present a higher tensile strength and presented a lower specific tensile strength. However, the increased tensile strength of the GFF/PP composite foams indicated an improvement in the impregnation effect and interfacial bonding. The SCN decreased the viscosity, which benefited the direct in-mold impregnation of the GFF. Increasing the temperature can improve the interfacial bonding, but it also influenced the foaming and thus led to a decrease in the tensile strength. According to the temperature distribution, the samples from different positions in the mold cavity had different properties.

## 1. Introduction

Continuous fiber reinforced thermoplastics polymer composites (CFRTPC), as a typical representative of lightweight materials, have been widely developed and applied in the fields of aerospace, automobile transportation, the chemical industry, electronic appliances, construction, home furnishings, sporting goods, etc. [1,2,3,4]. CFRTPCs are composed of a thermoplastic resin matrix, continuous fibers, and some additives. The resin matrix is distributed between the fibers to form a continuous phase, to fix the fibers and transmit loads at the same time. In comparison with short or long fiber reinforced thermoplastic composites, CFRTPCs have superior mechanical properties. However, the production methods for CFRTPCs’ parts are difficult, particularly realizing a suitable interfacial bonding between the matrix and the continuous fibers.

Injection molding is an efficient process to produce CFRTPCs’ parts. Generally, the production of injection molded CFRTPC parts requires two processes: prepreg preparation and insert injection molding [5,6,7,8]. The integrated injection molding process can finally obtain lightweight and strong parts and allow complex shapes. However, the prepreg preparation is complicated. The prepreg materials are usually sheets and strips, which are processed by the impregnation of woven fabrics or unidirectional fibers with a thermoplastic matrix. There are various preparation techniques for the prepregs, such as solution impregnation, melt impregnation, powder impregnation, pultrusion, compression, and in-situ impregnation [9,10]. Therefore, the chosen preparation process for prepregs affects the molding cycle and increases energy consumption [11,12,13]. Wang et al. [14] proposed a successful method that can realize the production of single-polymer composites whose matrix and fiber reinforcement are from the same polymer. They directly set the fabrics as an insert in the mold cavity and then applied the insert injection molding process. The influences of the injection molding process parameters, including the temperature, pressure, and time, were especially discussed [14]. Moreover, in order to improve the permeability, they presented an insert injection molding method whereby the inserted fabric was fixed in the middle of the mold cavity using clamping force [15]. Direct in-mold impregnation of PP fabrics by PP melts was realized successfully. Studer et al. [16] investigated the direct impregnation of carbon fiber fabrics with thermoplastic, on an injection molding machine. To improve the impregnation quality, polypropylene (PP) with a low viscosity, ranging from 15 to 50 Pa·s, was used. The direct impregnation of glass fabrics with low-viscosity polyamide (PA) through injection molding has been investigated [17]. The influence of temperature, pressure, and impregnation time was investigated. A high production rate and reliability of the process are possible, which makes it more attractive for the automotive sector. However, the impregnation time of 5 min is still much too long for injection molding. The impregnation rate of the plastic resin to the continuous fibers is usually low, because the reinforcement phase and the matrix are not of the same material [18], which easily causes pores and incomplete impregnation. Especially for the insert injection molding, the fabric is usually inserted on the cavity surface, where the polymer melts cool rapidly. The rapidly increase in viscosity will lead to incomplete impregnation [19], and thus it faces the problem of poor interfacial bonding. Therefore, how to improve the impregnation rate during a very short injection molding time, is a major problem to be solved [20,21,22]. Jeong et al. [23] investigated the direct impregnation of carbon woven fabric with a PP melt in injection molding. Particularly, they applied an aluminum mesh between the woven fabric and mold surface to improve the impregnation rate. A significant improvement in the tensile strength proved the feasibility of this process. Tröltzsch et al. [24] investigated the direct melt impregnation of unidirectional glass fiber tapes in the injection molding process. The injection was delayed by 30 s, so that the fiber tapes could be preheated in the cavity. Werlen et al. [25] presented a novel tool for thermoplastic compression resin transfer molding combined with injection molding. A variotherm process was successfully implemented with glass fabric and PA6, to produce plates at different temperatures. However, the cycle time should be reduced to under 20 min for industrial applications.

In contrast to solid thermoplastic composites, microcellular foamed polymer materials have advantages such as being lightweight, having a good dimensional stability, thermal insulation, sound insulation, shockproof buffering, high specific strength, and low price [26,27,28,29]. The foaming process follows four steps. The first is to introduce gas into the plastic melt to establish a homogeneous system of gas and polymer, to form bubble nuclei, and then the bubble nuclei grow into cells, which are fixed and shaped. There are four main preparation methods for thermoplastic foaming: extrusion foaming, injection foaming, autoclave foaming, and molding foaming [30,31,32]. In the microcellular injection molding process, the polymer melt is mixed with supercritical fluid (SCF) carbon dioxide or nitrogen in the injection barrel to form a single-phase polymer–gas solution, and then the polymer–gas solution is injected into the mold to form parts with numerous microscale cells. However, the foamed products usually have low mechanical strengths due to the easy diffusion of cracks around the cells. This limits its application in some high-strength and low-weight fields [33]. The mechanical properties can be improved by adding reinforcing materials. The mixing of the polymer matrix, reinforcement, and SCF can form a composite foam, whereas the reinforcement usually comes from short or long fibers. The interaction between the polymer matrix, reinforcement, and SCF determines the specific mechanical strength of the composite foam [34]. How to improve the comprehensive mechanical properties of composite materials with the coexistence of the polymer matrix, reinforcement, and SCF, has also become a major research direction [35,36]. The mechanical properties, normalized by weight ratio, of the fiber reinforced composite foams can be increased by optimizing the fiber dispersion and microcellular structures [37]. However, for the continuous fiber reinforced thermoplastic foams, insert injection molding has to be applied. Kasemphaibulsuk et al. [38] developed sandwich panels that consisted of a discontinuous glass fiber reinforced PP composite foam core and continuous glass fiber reinforced PP laminate skins. The discontinuous glass fiber reinforced PP with supercritical nitrogen (SCN) was injection molded onto the laminate skins, and then the foam core was formed. It suggested that the addition of continuous glass fiber reinforced PP laminate increased the flexural strength of the composite foam significantly. However, the laminate has to be manufactured before the injection molding. Wang and Chen [39,40] applied insert-microcellular injection molding to realize the direct in-mold impregnation of PP fabric by PP/SCN solution. The fabric was set in the middle of the cavity, and the self-reinforced mechanism realized a better interfacial bonding between the PP fabric and the PP melt. A sandwiched PP single-polymer composite foam with enhanced specific mechanical strength was obtained.

In this study, we proposed an integrated injection molding technique, by combining microcellular injection molding and insert injection molding. The glass fiber fabric (GFF) can be directly set on the surface of the mold cavity and then impregnated by the PP/SCN solution, using insert-microcellular injection molding. Since the PP/SCN solution has a lower viscosity than the PP melt, it is supposed to be beneficial to impregnation. The foaming process provides pressure that can also improve impregnation. On the other hand, the preparation of intermediate laminates, or sheets, can be avoided, and a one step process to net-shaped products with strong mechanical properties of continuous fibers can be realized. We investigated the direct in-mold impregnation of GFF by microcellular injection molded PP/SCN solution. Two types of GFFs were applied. The effect of the injection temperature of the PP/SCN solution on the impregnation of the continuous glass fibers and the interfacial bonding was especially analyzed. The morphological and tensile properties of the GFF reinforced PP composite foams were characterized in comparison with the pure PP foams, unfoamed PP, and unfoamed GFF/PP composites. The tensile properties of the samples at different positions in the mold cavity was also analyzed. The results demonstrated the improvement of the in-mold impregnation of GFF through the insert-microcellular injection molding process.

## 2. Materials and Methods

### 2.1. Materials

**Figure 1:**
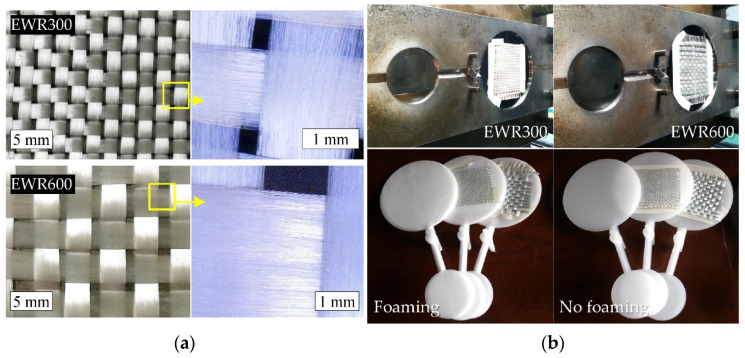
Photos of the glass fabrics (**a**), the mold cavity with inserted glass fiber fabrics and the prepared samples (**b**).

PP particles (model number: E02ES, Sinopec Zhenhai Refinery, Zhenhai, China) with a density of 0.9 kg/m^3^ at room temperature were used as the matrix. Two types of GFF (EWR300, Changzhou KPSD New Materials Co., Ltd. (Changzhou, China); and EWR600, Zhejiang Weitong Composite Co., Ltd., Tongxiang, China) were selected as the reinforcement materials. Table 1 presents the main information on the GFFs. Figure 1a shows the two plain fabric weaving structures and their relative fiber bundles.

### 2.2. Preparation

The GFF reinforced PP composite foam samples were prepared by a combination of insert injection molding and microcellular foam injection molding. The injection molding machine (MA1600IIS, Haitian Group, Ningbo, China), equipped with a microcellular foaming system (Beijing CHN-TOP Machinery Group Co., Ltd., Beijing, China), was applied. Supercritical nitrogen (SCN) was used as a physical blowing agent. Figure 1b shows the mold cavity with the GFFs inserted, and the prepared samples. A disc geometry with a diameter of 100 mm and a thickness of 3 mm was applied. The GFF was firstly cut into a square (× mm) and then placed into the mold cavity as an insert. Paper tapes were used to fix the fabric. The PP melt with SCN solutions was injected into the mold cavity to fill the cavity and penetrate the fabric. After the cooling stage, the composite foam with GFF was finally obtained. Unfoamed samples were also prepared for comparison. The process parameters of the injection molding are shown in Table 2. Three injection temperatures (230, 240, and 250 °C) were used, while the other process parameters were kept the same. The injection velocity, from 50 to 40 mm/s, as a function of the screw displacement, 12 mm, was applied, and the relative injection pressure was between 70 and 60 bar. It is noted that the exact pressure was not up to the setting injection pressure. Due to the foaming process, packing pressure was not applied in the microcellular injection molding. The SCN flowrate was 0.5 kg/h, the SCN injection pressure was 125 bar, and the dosage time was 0.5 s. The cooling time was 30 s. For comparison, the conventional injection molding was conducted under the same conditions, except a packing pressure of 30 bar and packing time of 3 s were applied.

### 2.3. Numerical Simulation

**Figure 2:**
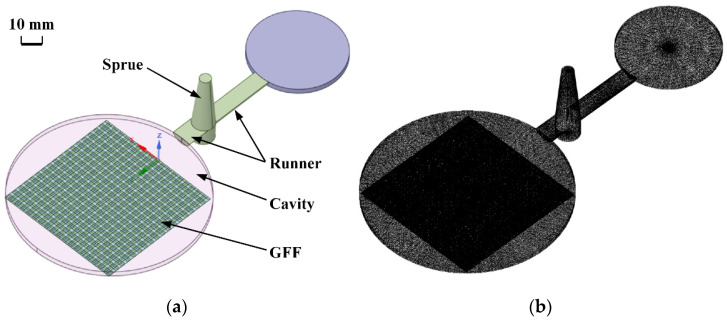
3D model of the insert injection molding with GFF EWR300 (**a**), and the mech model (**b**).

The in-mold impregnation process of GFF EWR300 was simulated by Moldex 3D (CoreTech System Co., Ltd., Tainan, Taiwan). The 3D model is shown in Figure 2. The sprue, runner, mold cavity, and one layer of GFF EWR300, according to the exact experimental sample, were all included in the model. The fabric structure was set according to the exact warp-weft structure of the EWR300, but the fiber bundles were kept in ensemble. The thickness of the GFF was set as 0.3 mm, and the deformation of the GFF during the molding process was not considered. The material of the GFF was typical glass, and the polymer material was PP. These typical materials can all be found in the material database of the software. The total mesh number was 1,796,956. For comparison, the injection molding processes (foaming and unfoaming) without GFF were also simulated. The injection molding parameters are all shown in Table 2.

### 2.4. Weight Measurement and Tensile Testing

The injection molded samples were cut into several tensile bars by a metallic cutter according to the specimen type of 1BA (ISO 527-2), with lengths of 80 mm and widths of 10 mm. For each sample without fabric, six tensile samples could be cut. For each sample with fabric, four samples were cut, in order to keep the same number of fiber bundles in one tensile bar. Two fiber bundles could be kept on the tensile sample with the GFF of EWR300, and one fiber bundle could be kept on the tensile sample with the GFF of EWR600. In order to avoid fiber debonding during the cutting, continuous fibers were cut off with knives in advance. Four tensile samples of each group were used for the tensile testing. The weight of each tensile sample was measured by an electronic balance (FR124CN, Ohaus Corporation, Shanghai, China) with an accuracy of 0.0001 g. The tensile properties of the samples were measured by a universal testing machine (WDT-W, Chengde Precision Testing Machine Co., Ltd., Chengde, China) according to ISO 527-1: 1993. The tensile speed was 5 mm/min. Each test was performed at room temperature. The ultimate tensile strength and the elongation at break were recorded. The sample weight combined with the sample volume was used to calculate the specific tensile strength.

### 2.5. Morphological Observation

The disc samples were put on a glass platform of an instrument (Stress Viewer R5.1) supported by Moldex 3D, CoreTech System Co., Ltd. (Zhubei City, Taiwan), and the pictures were taken from above by a camera (EOS M3, Canon Inc., Tokyo, Japan). Through the backlighting of the instrument, the microcellular structure and the plain weaving structure of the GFFs in the disc samples can be observed. The pictures of the tensile samples were also taken by the camera for comparison. Especially, the cut sections of each group of tensile samples were photographed with a large magnification, thus the microcellular foamed structure and the impregnation effect of the fabric could be analyzed. Pictures of the fractured tensile samples after testing were also taken, to know their tensile properties.

The image analysis software ImageJ (National Institute of Mental Health, Bethesda, MD, USA) was used for the quantitative analysis of the cell size and cell density of the foamed samples. The cell density (cells/cm^3^) was calculated using (N/A)^3/2^, where N is the cell number and A is the area where the cell number was counted.

## 3. Results

### 3.1. Impregnation Process

**Figure 3:**
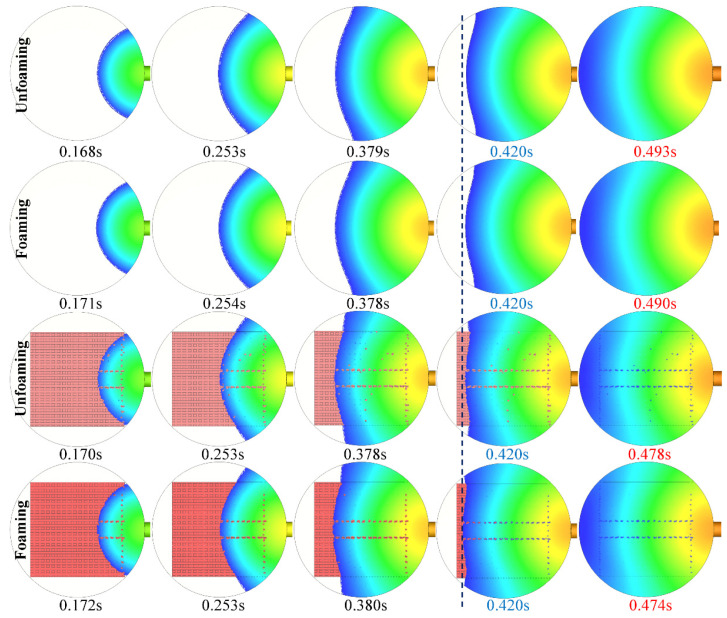
Numerically simulated conventional injection molding (unfoaming), microcellular injection molding (foaming), insert injection molding (unfoaming), and insert-microcellular injection molding (foaming). The numbers in red color are the total filling time of the mold cavity. The 0.420 s in blue color indicates an example for comparison of the filling melt front.

Figure 3 illustrates the numerically simulated processes, including conventional injection molding (unfoaming), microcellular injection molding (foaming), insert injection molding (unfoaming), and insert-microcellular injection molding (foaming). The impregnation process of the GFF by the PP melt took 0.478 s, which is a little bit longer than the impregnation time (0.474 s) of the GFF by the PP/SCN. As the dashed line in Figure 3 shows, the PP/SCN solution moved further than the PP melt at the same time. This indicates that the foaming process benefits the impregnation of the GFF, due to the lower viscosity of the PP/SCN and the pressure generated by the cellular foaming.

### 3.2. Morphological Properteis

#### 3.2.1. Apparent Morphology

**Figure 4:**
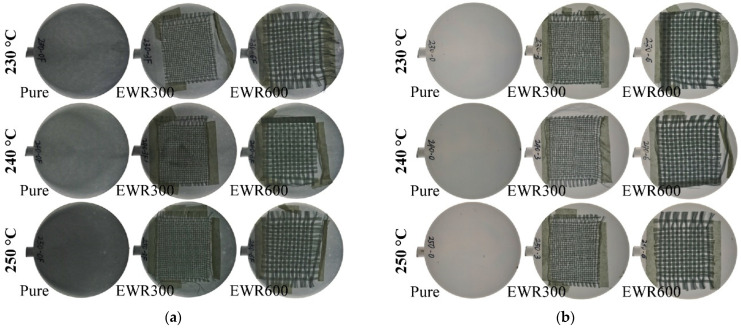
Pictures of the foamed samples (**a**) and unfoamed samples (**b**) under backlighting.

Figure 4 illustrates pictures of the disc samples, which were taken by the camera with a background of LED lighting. Under the backlighting, the plain weaving structure of the fabrics can be clearly seen due to the semitransparent property of PP. The fiber bundles were not tight because they were cut. The bundles of samples with EWR600 GFF present were tighter than those samples with EWR300 GFF, due to there being more fibers in one bundle. In comparison with the unfoamed samples, the foamed samples appeared darker, because of the microcellular structures inside the samples. At higher injection temperatures, the foamed samples appeared darker, indicating microcellular structures with higher cell densities and amounts.

**Figure 5:**
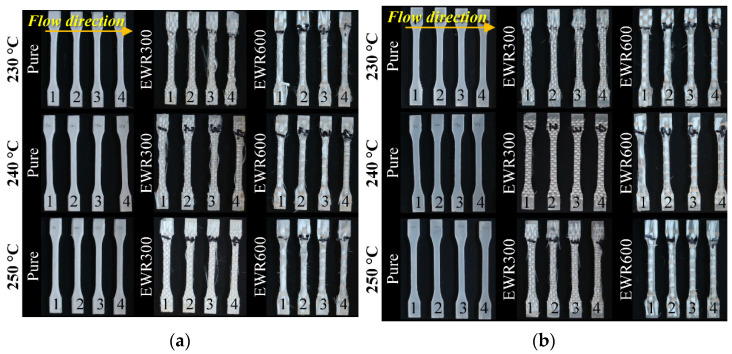
Pictures of the foamed (**a**) and unfoamed (**b**) tensile samples. The numbers of 1, 2, 3, and 4 indicate the number for each type of samples.

**Figure 6:**
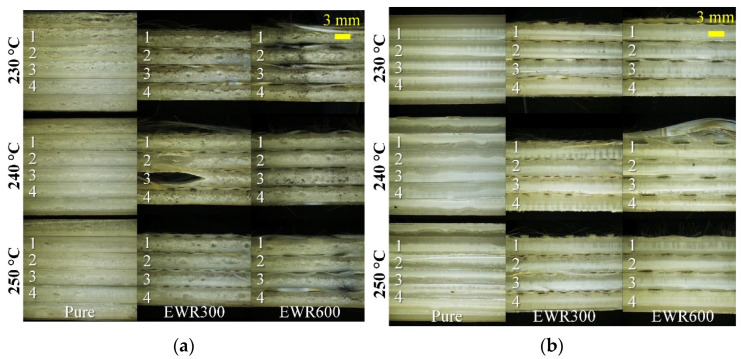
Pictures of the cut thick sections of the foamed tensile samples (**a**) and unfoamed tensile samples (**b**). The numbers of 1, 2, 3, and 4 indicate the number for each type of samples.

Figure 5 shows the tensile samples which were cut from the disc geometry samples. Figure 6 shows photos of the cut sections of the tensile samples. In comparison with the unfoamed samples, the foamed samples showed a typical sandwiched microcellular structure: the middle layer with big cells, transition layers with small cells, and skin layers with frozen unfoamed skin and filamentous cells. However, due to the existence of a layer of GFF on the top of the samples, the microcellular structure was influenced. It is seen that the samples with GFFs had more and bigger cells than those without GFFs. In comparison with the foamed samples with EWR300 GFF, the foamed samples with EWR600 GFF had better microcellular structures (smaller cell size and higher cell density) due to the flatter weaving structure. At the injection temperatures of 240 and 250 °C, the foamed samples showed better microcellular structures in comparison to the samples prepared at 230 °C. However, the effect of the injection temperature on the interfaces between the matrix and the glass fabric was not clear. Disbonding fiber bundles can be seen, especially for the part of the fiber bundles which were buckled by the crossed bundles. In addition, in comparison with the unfoamed samples, the impregnation of the glass fabrics was not improved by the foaming process. The viscosity could be decreased by the SCN solution, however, the PP/SCN solution experienced the process with rapid decreases in pressure and temperature. Thereafter, the impregnation was not improved significantly in the short cycle time, even at a higher injection temperature of 250 °C.

#### 3.2.2. Fracture Morphology

**Figure 7:**
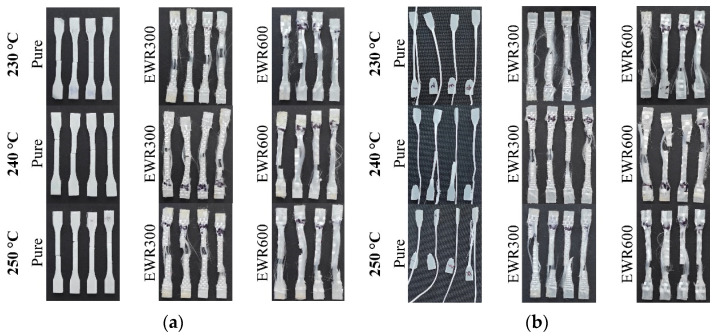
Pictures of the foamed (**a**) and unfoamed (**b**) samples after tensile testing.

**Figure 8:**
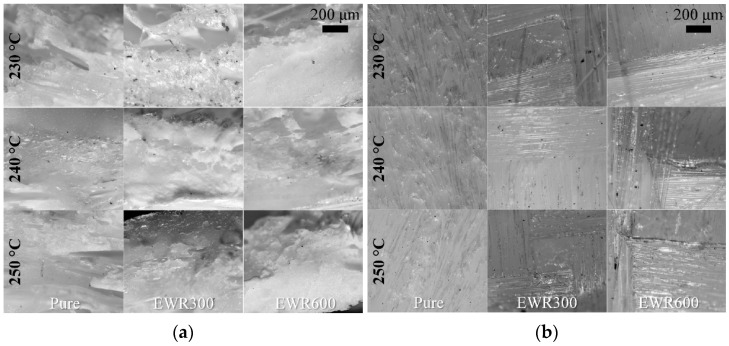
Microscopy photos of the fracture sections (**a**) and the surface sections (**b**) of the tensile samples with foaming.

The fracture morphologies of the samples are shown in Figure 7 and Figure 8. The foamed samples without glass fabrics showed a typical brittle fracture, and the pure unfoamed PP samples showed great ductility. The fracture of the samples with fabrics included the disbonding of the fiber bundles and the breaking of the matrix. It showed bad interfacial adhesion between the fabric and the matrix. There was no difference in the interfacial bonding between microcellular injection molding and normal injection molding. Figure 8a shows that the cell size decreased when using glass fabrics. Smaller cells and higher cellular densities can be seen at the fracture sections of the foamed samples with EWR600 GFF. Figure 8b illustrates the obvious silver streak and swirl which are typical surface defects of microcellular samples. There were obvious imprints of glass fibers on the surfaces of the samples with EWR300 GFF and EWR600 GFF. Little glass fibers were left on the samples, which indicates poor interfacial adhesion.

#### 3.2.3. Cell Morphology

**Figure 9:**
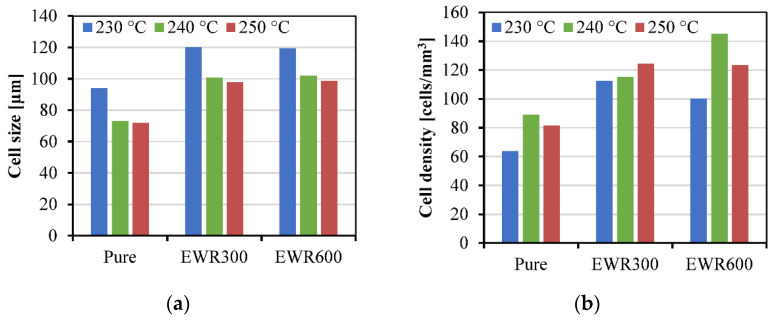
The average cell size (**a**) and cell density (**b**) of the tensile samples with foaming.

**Figure 10:**
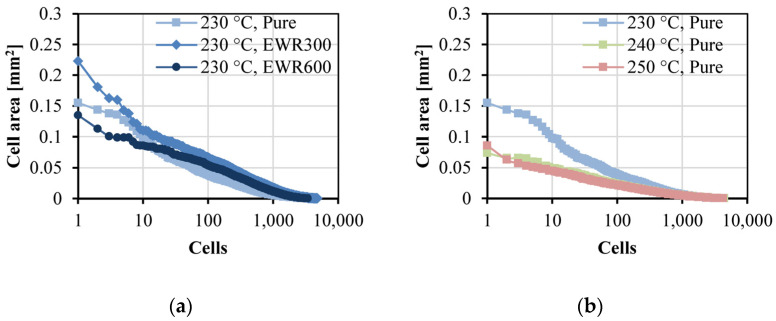

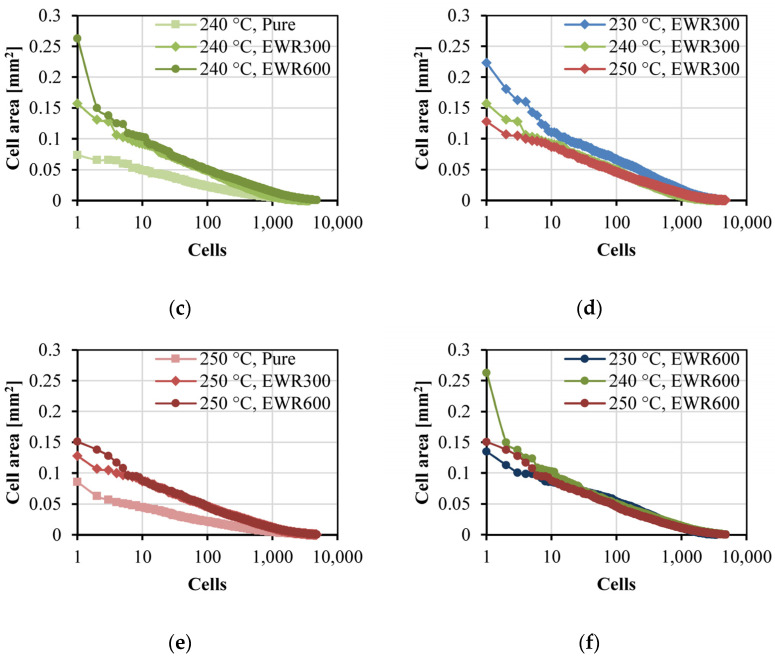
Cell areas (arranged in descending order) in the section area of the foamed samples. The curves for the comparison of the different samples are respectively shown in (**a**), (**c**), and (**e**), and the curves for the comparison of the samples at different temperatures are respectively shown in (**b**), (**d**), and (**f**).

The results for cell size and cell density are shown in Figure 9 and Figure 10 shows the cell areas (arranged in descending order) in the section area of the foamed samples. The largest average value of cell size was 120 μm, that was for the foamed sample with EWR300 GFF prepared at the injection temperature of 230 °C, and the smallest average value of cell size was 73 μm. The increase in injection temperature led to a smaller cell size. This is because the higher temperature reduces the melt strength. The addition of GFF significantly increased the cell size and cell density. This is due to the increased shear rate caused by the inserted fabric, and the increased shear rate can lead to an increase in cell nucleation. Moreover, glass fibers can lower the energy barrier and improve cell nucleation. However, the mechanism of cell nucleation and cell growth in this insert-microcellular injection molding process is different from in the traditional microcellular injection molding for inorganic filler-reinforced polymer composites. Due to the GFF consisting of continuous glass fibers, not like the inorganic fillers compounded with the polymer in the barrel, the energy barrier was only established at the fabric interfaces during the impregnation process, but the gas solubility was not influenced. During the direct in-mold impregnation process, the PP/SCN solution filled into the cavity and then permeated the GFF, the heat transfer occurred between the continuous glass fibers and the PP/SCN solution. Thus, the energy absorption benefited cell nucleation and cell growth. However, there was no significant difference in the cell sizes of the samples with EWR300 and EWR600. This indicates that the interfacial energy established by the EWR300 and EWR600 was similar. In addition, in comparison with the foamed samples with EWR300, the cell densities of the foamed samples with EWR600 decreased at the temperature of 230 °C, increased at the temperature of 240 °C, and did not change a lot at the temperature of 250 °C. This indicates that the influence of the temperature and the fabric structure on the cell density was complicated. The highest cell density was 145 cells/mm^3^, that was for the foamed sample with EWR600 GFF prepared at the injection temperature of 240 °C. Consequently, the inserted EWR600 GFF can lead to a better microcellular structure (smaller cell size and higher cell density), but a suitable temperature should be used.

### 3.3. Tensile Properties

**Figure 11:**
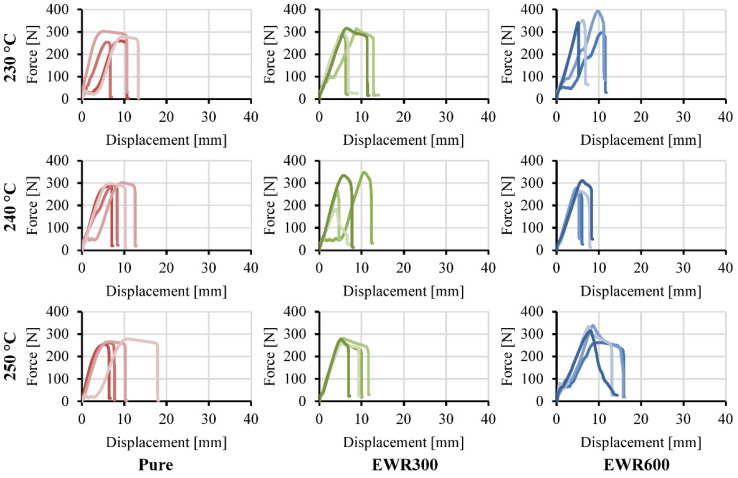
Force-displacement curves of the tensile foamed samples prepared at different injection temperatures. The different color shows different samples.

**Figure 12:**
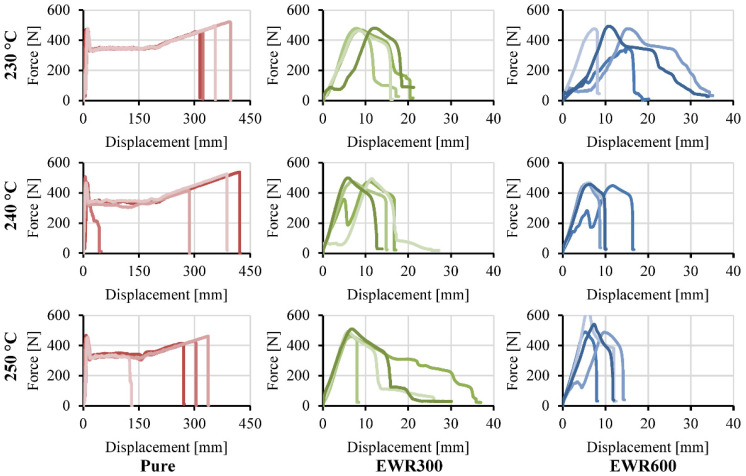
Force-displacement curves of the tensile unfoamed samples prepared at different injection temperatures. The different color shows different samples.

Force-displacement curves of the tensile foamed and unfoamed samples are shown in Figure 11 and Figure 12, respectively. Clamp slippage caused the fluctuation and stagnation of force at the beginning of some of the curves. The foamed samples show lower breaking strengths and elongations at break in comparison with the unfoamed samples. The bonded fabrics improved the rigidity, but this was not significant due to the weak interfacial bonding. Table 3 gives the weight, tensile strength, specific tensile strength, and elongation at break of the tensile samples.

#### 3.3.1. Effect of Injection Temperature

**Figure 13:**
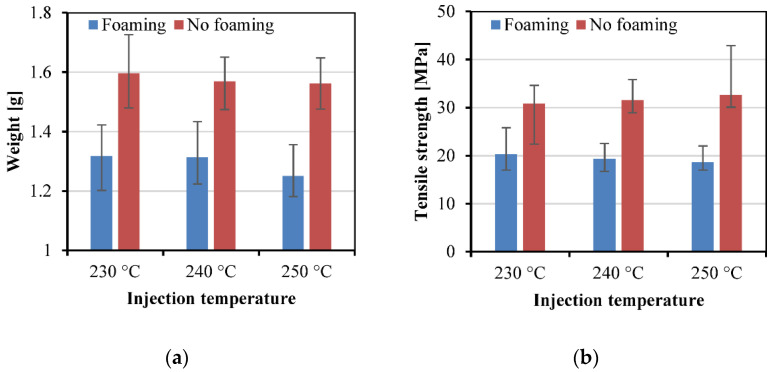

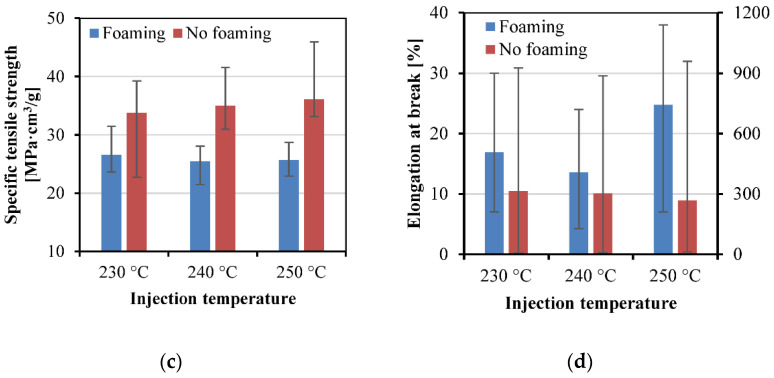
Weight (**a**), tensile strength (**b**), specific tensile strength (**c**), and elongation at break (**d**) of the samples prepared at different injection temperatures.

Figure 13 shows the effect of the injection temperature on the weight, tensile strength, specific tensile strength, and elongation at break of the samples. The foamed samples had lower weight, tensile strengths, specific tensile strengths, and elongations at break in comparison with the unfoamed samples. Higher injection temperatures led to lower weights for both foamed and unfoamed samples. The tensile strengths and specific tensile strengths of the foamed samples decreased with increasing injection temperature, however, those of the unfoamed samples increased. This indicates that a higher injection temperature benefited microcellular foaming, thus the strength and even the specific tensile strength decreased. The strengths of the unfoamed samples increased, and the elongations at break decreased, with increasing injection temperature, which might be due to the increased crystallization at higher temperatures.

#### 3.3.2. Effect of Sample Structure

**Figure 14:**
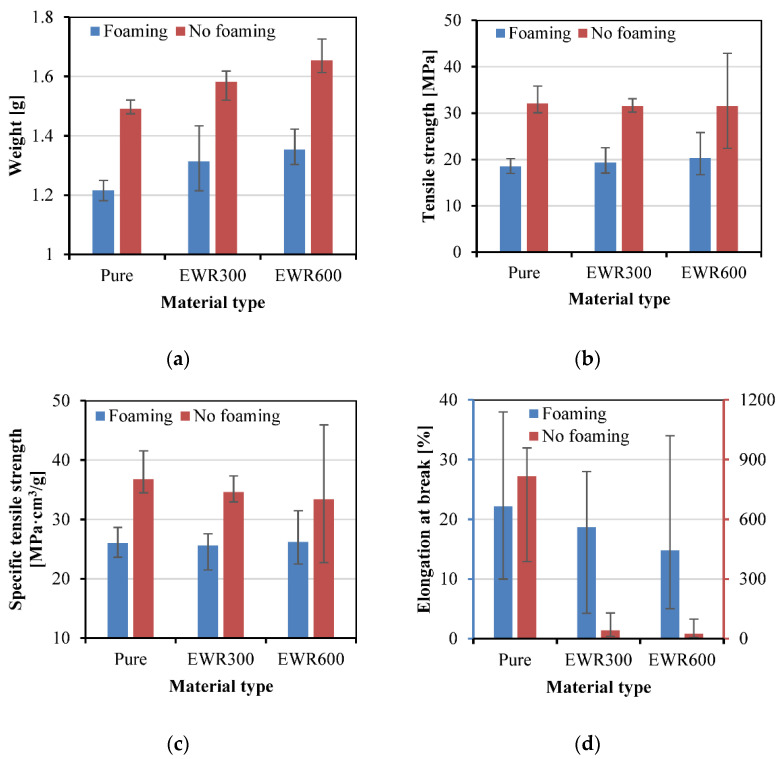
Weight (**a**), tensile strength (**b**), specific tensile strength (**c**), and elongation at break (**d**) of the samples without and with GFF reinforcement.

Figure 14 shows the effect of sample structure on the weight, tensile strength, specific tensile strength, and elongation at break of the samples. The composite of GFF significantly increased the weight of the samples due to the much higher density of the GFF compared to the PP. The samples with EWR600 GFF had higher weights than the samples with EWR300 GFF due to the higher areal density. The average tensile strengths of the unfoamed samples were not affected by the GFF, and the average specific tensile strengths decreased when the GFF was added. Whereas, the big error range of the tensile strength indicates that there was an increase when the EWR600 GFF was used in the unfoamed samples, but the interfacial bonding between the fabric and the matrix without foaming was unstable. This result shows the difficulty of using continuous glass fiber in the reinforcement role in the insert injection molding, due to the heterogeneity between the glass fiber and the polymer. However, for the foamed samples, the average tensile strengths increased by 4% and 10%, respectively, when the EWR300 and EWR600 were added. By comparing the maximum values, the increase in tensile strength could be up to 12% and 28%, respectively. The increase in tensile strength proves that the composite of GFF with PP/SCN solution can improve the mechanical strength of the foamed composite samples. In comparison with the pure PP melts, the PP/SCN solutions can have a lower viscosity and thus can improve the impregnation of the GFF. In addition, compared to the EWR300 GFF, the fabric structure of EWR600 GFF benefits the interfacial adhesion between the fabric and the matrix. Due to the higher fiber volume fraction and better cellular structure, the EWR600 GFF/PP composite foams had higher specific tensile strengths than the EWR300 GFF/PP composite foams. However, due to the high density of the GFF, the specific tensile strengths of the GFF/PP composite foams increased little in comparison with the pure PP foams. The addition of GFF led to a significant decrease in the elongations at break of the unfoamed samples. The elongations at break of the foamed samples also decreased due to the addition of GFF, and decreased more when using the EWR600 GFF in comparison with the EWR300 GFF.

#### 3.3.3. Effect of Position

**Figure 15:**
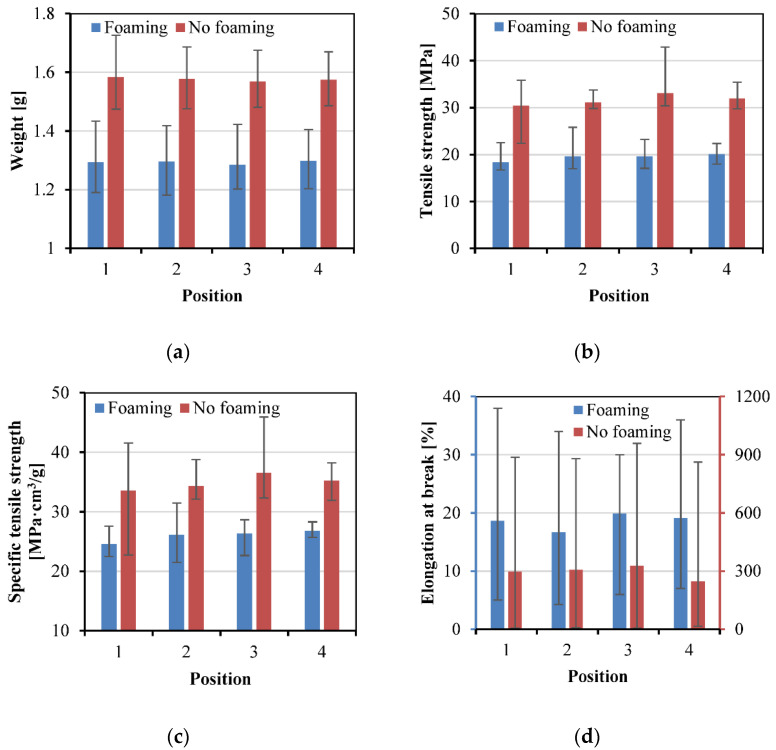
Weight (**a**), tensile strength (**b**), specific tensile strength (**c**), and elongation at break (**d**) of the tensile samples cut from different positions in the disc sample.

Figure 15 shows the weight, tensile strength, specific tensile strength, and elongation at break of the samples at different positions of the mold cavity. Generally, the pressure and temperature are higher at the position near the gate. The higher pressure and temperature can improve the impregnation and thus lead to a higher interfacial adhesion. However, the results of the tensile strength and specific tensile strength showed a only small increase from Position 1 (near the gate) to Position 3, and then a small decrease at Position 4 (far from the gate). This is due to the disc geometry of the mold cavity leading to higher temperatures and pressures close to the position between the center and the end of the mold cavity. In addition, the effect of position on the sample weight and elongation at break was not significant.

## 4. Conclusions

The GFF/PP composite foam can be successfully manufactured through the direct in-mold impregnation of GFF by microcellular injection molding of PP/SCF solution. The morphological characterization showed that the microcellular structure was influenced by the addition of GFF. Energy absorption by the addition of GFF benefited cell nucleation and cell growth, so the GFF/PP composite foam, in comparison with the PP foam, had a larger cell size and a higher cell density. Compared with the EWR300, the EWR600 GFF can lead to a better microcellular structure (smaller cell size and higher cell density), but a suitable temperature should be used. The breaking morphology showed poor interfacial bonding for all types of samples due to the heterogeneity between the PP and the GFF. The tensile strength of unfoamed PP was not significantly increased by adding the GFF, which proved the poor interfacial adhesion between the PP and GFF. The specific tensile strength was decreased due to the increased weight by adding the GFF. However, the tensile strength of the GFF/PP composite foam was higher than that of the PP foam. The average tensile strength of the EWR600 GFF/PP composite foam was 10% higher than that of the PP foam. This indicated that the permeability was improved by the PP/SCF solution with a low viscosity. The specific tensile strength of the EWR600 GFF/PP composite foam was higher than that of the EWR300 GFF/PP composite foam due to the higher fiber volume fraction and the better cellular structure. However, the specific tensile strength of the GFF/PP composite foam was not higher than that of the PP foam due to the high density of the GFF. Increasing the injection temperature increased the tensile strengths and specific tensile strengths of the unfoamed samples. However, the tensile strengths of the foamed samples was not increased, because the microcellular foaming was also improved by increasing the injection temperature. The geometry of the mold cavity also influenced the impregnation of the GFF. The samples at the position between the center and the end of the mold cavity had higher tensile strengths and specific tensile strengths. That is because the PP melts with a higher temperature and lower viscosity located at the position between the center and the end of the disc geometry cavity.

This work has demonstrated the micro-foaming process has an improved effect on the impregnation of a continuous fiber fabric with a polymer melt. However, the limited increase in tensile strength indicates that the direct in-mold impregnation of heterogeneous fabrics by injection molded polymer melt with SCF still needs the optimization of the injection molding process window and some assisted techniques, such as preheating and insert tools. Further understanding of the impregnation mechanism, and the definition of the processing window as a function of matrix material and reinforcement fabric, will be our future work.

## Figures and Tables

**Table 1:** Material information of the glass fiber fabrics.

Brand	Materials	Areal Density (g/m^2^)	Breaking Strength (≥N/25 mm)		Number of Bundles (Threads/cm)		Woven Type	Thickness (mm)
			Warp	Weft	Warp	Weft		
EWR300	E-glass	300 ± 15	1200	1200	5	5	Plain	0.3
EWR600	E-glass	600 ± 30	1800	1800	2.5	2.5	Plain	0.6

**Table 2:** Process parameters of the injection molding processes.

Process Parameters	Microcellular Injection Molding	Conventional Injection Molding
Injection temperature (°C)	230, 240, and 250	230, 240, and 250
Injection velocity profile (mm/s)	50 → 40	50 → 40
Injection pressure profile (bar)	70 → 60	70 → 60
Velocity/Pressure switch-over	by screw position	by screw position
Packing pressure (bar)	n/a	30
Packing time (s)	n/a	3
Cooling time (s)	30	30
SCN injection flowrate (kg/h)	0.5	n/a
SCN injection pressure (bar)	125	n/a
SCN dosage time (s)	0.5	n/a

**Table 3:** Weight, tensile strength, specific tensile strength, and elongation at break of the tensile samples.

Process	Injection Temperature [°C]	Material Type	Weight [g]	Tensile Strength [MPa]	Specific Tensile Strength [MPa·cm^3^/g]	Elongation at Break [%]
Foaming	230	Pure	1.218 ± 0.012	18.263 ± 1.423	25.622 ± 2.221	20.250 ± 7.932
		EWR300	1.326 ± 0.001	19.888 ± 1.003	26.121 ± 1.204	20.000 ± 6.055
		EWR600	1.408 ± 0.022	22.690 ± 2.622	27.982 ± 3.108	10.500 ± 3.317
	240	Pure	1.232 ± 0.012	19.550 ± 0.564	27.107 ± 0.732	17.750 ± 6.850
		EWR300	1.418 ± 0.014	20.633 ± 2.857	25.390 ± 3.384	16.247 ± 10.411
		EWR600	1.318 ± 0.016	18.050 ± 1.526	23.980 ± 1.902	7.250 ± 3.862
	250	Pure	1.195 ± 0.011	17.710 ± 0.655	25.310 ± 0.800	28.500 ± 10.376
		EWR300	1.221 ± 0.006	17.710 ± 0.658	25.223 ± 0.944	19.250 ± 8.958
		EWR600	1.335 ± 0.022	20.310 ± 2.280	26.609 ± 2.593	26.500 ± 8.103
Unfoaming	230	Pure	1.510 ± 0.020	32.410 ± 1.717	37.092 ± 1.631	880.000 ± 30.735
		EWR300	1.590 ± 0.047	30.890 ± 0.466	33.912 ± 0.745	31.750 ± 16.358
		EWR600	1.690 ± 0.026	29.190 ± 4.565	30.242 ± 5.039	29.500 ± 46.336
	240	Pure	1.484 ± 0.008	33.100 ± 2.405	38.108 ± 2.938	848.000 ± 54.851
		EWR300	1.581 ± 0.007	31.920 ± 0.890	34.993 ± 0.815	38.250 ± 19.517
		EWR600	1.643 ± 0.008	29.710 ± 0.575	31.821 ± 0.585	20.750 ± 8.732
	250	Pure	1.480 ± 0.005	30.6625 ± 0.522	35.202 ± 0.712	720.250 ± 249.600
		EWR300	1.573 ± 0.012	31.590 ± 1.326	34.948 ± 1.929	58.500 ± 54.274
		EWR600	1.629 ± 0.014	35.500 ± 5.229	38.082 ± 5.589	22.750 ± 2.872

## Data Availability

The datasets generated and analyzed during the current study are available from the corresponding author on reasonable request.
